# In *Candida albicans* hyphae, Sec2p is physically associated with *SEC2* mRNA on secretory vesicles

**DOI:** 10.1111/mmi.12799

**Published:** 2014-10-14

**Authors:** David Caballero-Lima, Guillaume M Hautbergue, Stuart A Wilson, Peter E Sudbery

**Affiliations:** Department of Molecular Biology and Biotechnology, University of SheffieldWestern Bank, Sheffield, S10 2TN, UK

## Abstract

*Candida albicans* hyphae grow in a highly polarized fashion from their tips. This polarized growth requires the continuous delivery of secretory vesicles to the tip region. Vesicle delivery depends on Sec2p, the Guanine Exchange Factor (GEF) for the Rab GTPase Sec4p. GTP bound Sec4p is required for the transit of secretory vesicles from the trans-Golgi to sites of polarized growth. We previously showed that phosphorylation of Sec2p at residue S584 was necessary for Sec2p to support hyphal, but not yeast growth. Here we show that on secretory vesicles *SEC2* mRNA is physically associated with Sec2p. Moreover, we show that the phosphorylation of S584 allows *SEC2* mRNA to dissociate from Sec2p and we speculate that this is necessary for Sec2p function and/or translation. During hyphal extension, the growing tip may be separated from the nucleus by up to 15 μm. Transport of *SEC2* mRNA on secretory vesicles to the tip localizes *SEC2* translation to tip allowing a sufficient accumulation of this key protein at the site of polarized growth.

## Introduction

*Candida albicans* hyphae show extreme polarized growth from their tip (Soll *et al.*, 1985). This polarized growth requires a flow of post-Golgi secretory vesicles along actin cables. These deliver the additional membrane and enzymes that manufacture and re-model new cell wall required for tip extension (Sudbery, 2011; Caballero-Lima *et al.*, 2013). Research in *Saccharomyces cerevisiae* has shown that at sites of polarized growth the vesicles are tethered to the exocyst, an octomeric protein complex attached to the inner surface of the plasma membrane (Terbush and Novick, 1995; Terbush *et al.*, 1996). The vesicle-associated Rab GTPase Sec4p in its GTP-bound form is required for the late stages of the secretory pathway (Walworth *et al.*, 1989). It mediates the tethering of vesicles with the exocyst by its interaction with the exocyst component Sec15p (Guo *et al.*, 1999). The activating GEF of Sec4p is Sec2p, which is also vesicle associated, but released to the cytosol upon vesicle tethering to the exocyst (Walch-Solimena *et al.*, 1997; Medkova *et al.*, 2006; Novick *et al.*, 2007). Thus there are vesicle-associated and cytosolic pools of Sec2p. At the tip of *C. albicans* hyphae both Sec2p and Sec4p are localized to a subapical spot that is similar to the Spitzenkörper of filamentous fungi (Bishop *et al.*, 2010; Jones and Sudbery, 2010). Localization of Sec2p to this Spitzenkörper is dependent on phosphorylation at S584 by Cdk1p (Bishop *et al.*, 2010) In contrast to the localization of Sec2p and Sec4p, the exocyst components localize to a surface crescent (Bishop *et al.*, 2010; Jones and Sudbery, 2010).

An important aspect of protein localization in diverse cell types cells is the asymmetric localization of the encoding mRNA (Shahbabian and Chartrand, 2012; Xing and Bassell, 2013). A well-studied example in *S. cerevisiae* is the localization of the mRNA encoding Ash1p to daughter buds (Long *et al.*, 1997). This process that involves four localization signals or ‘Zip codes’, three in the ORF and one in the 3′ UTR (Chartrand *et al.*, 1999). In this case the mRNA is present in an RNA–protein complex (RNP) in association with Puf6p, Khd1p, She2p, She3p and Myo4p (Bertrand *et al.*, 1998; Boehl *et al.*, 2000; Long *et al.*, 2000; Takizawa and Vale, 2000; Irie *et al.*, 2002; Gu *et al.*, 2004). Khd1p and Puf6p repress *ASH1* translation during transport (Irie *et al.*, 2002; Gu *et al.*, 2004; Paquin *et al.*, 2007). The repressive effect of Khd1p is reversed by phosphorylation by the casein kinase Yck1p. She2p is an RNA-binding protein that shuttles between the nucleus and cytoplasm. She2p is loaded co-transcriptionally onto *ASH1* mRNA and forms a complex with a nuclear proteins Loc1p and Puf6p, (Shen *et al.*, 2010; Shahbabian *et al.*, 2014). Puf6p also binds at least 40 other transcripts in a She2p-dependent fashion, including mRNAs destined to transported to the bud. Myo4p, is a class V myosin that mediates the movement of RNPs along actin cables. She3p is the bridging protein that links She2p and Myo4p. She3p is also an RNA-binding protein so that recognition of *ASH1* mRNA and its incorporation of into a ribonucleoprotein (RNP) complex requires both She2p and She3p (Müller *et al*., 2011). The efficient transport of *ASH1* mRNA to daughter buds requires the polarized growth machinery since mutations affecting a wide range of polarity components, such as Sec4p, Cdc42p and exocyst components, reduces or abolishes *ASH1* mRNA localization (Aronov and Gerst, 2004). Bud-directed RNPs associate with the cortical ER and their transport is dependent on genes required for cortical ER segregation such as *MYO4* and *SHE3* (Aronov *et al.*, 2007). Consistent with the idea that RNPs are co-transported with the cortical ER to the daughter bud, She2p binds to tubular ER membranes *in vitro* (Shen *et al.*, 2010; Genz *et al.*, 2013).

Microarray experiments have demonstrated that many other mRNAs besides *ASH*1 can also be associated with She3p (Shepard *et al.*, 2003). In a separate study it was shown that mRNAs that encode polarity proteins, including Sec4p are themselves transported to the sites of polarized growth in a She3p-dependent fashion (Aronov *et al.*, 2007). These mRNAs colocalize at the tip of daughter buds with the proteins they encode. In a *she3* mutant mRNA protein localization is reduced but not abolished. Thus, mRNA localization increases the efficiency of protein localization, but is not essential. *ASH*1 is also asymmetrically localized in *C. albicans* (Inglis and Johnson, 2002)*,* and it has been shown that a number of mRNAs are asymmetrically localized in a She3p-dependent fashion (Elson *et al.*, 2009). This She3p-dependent set of mRNAs did not include any of the proteins that have been shown to localize to the hyphal tip; and apart from *ASH*1, there was only limited overlap with the set of genes that is associated with She3p in *S. cerevisiae*. *C. albicans* lacks an obvious She2p homologue. In view of the finding that She3p can recognize and bind *ASH1* mRNA, it is possible that She3p is solely responsible for recognizing and binding to *ASH1* mRNA in *C. albicans.*

The tip of *C. albicans* hyphae can be a considerable distance from the nucleus. For example, before nuclear migration into the germ tube, which precedes mitosis, the tip may be up to 15 μm from the nucleus. In contrast the bud tip of a *S. cerevisiae* diploid cell is unlikely to be more than 5 μm from the nucleus. We hypothesized that it was likely that the strongly polarized distribution of proteins at the hyphal tip may be mediated by a corresponding asymmetric distribution of their encoding mRNAs. Such an idea is supported by electron micrographs in other fungal hyphae which show that the Spitzenkörper is rich in polyribosomes suggesting that it is the site of intense protein translation (Grove *et al.*, 1970). We reasoned that the mRNAs may be transported on secretory vesicles since *ASH*1 mRNA transport in *S. cerevisiae* is dependent on components of the late secretory pathway such as Sec4p and the exocyst and localization of mRNAs encoding polarized proteins is dependent on She2p, She3p and Myo4p. We show here that mRNA is present in the immunoprecipitate of Sec2p, but surprisingly, the only mRNA present is the mRNA encoding Sec2p itself. Cell fractionation experiments shows that Sec2p mRNA co-fractionates with secretory vesicles, moreover the interaction of Sec2p with its RNA is lessened in a C-terminal truncation and is abolished by the S584E phosphomimetic substitution. Thus, secretory vesicles are not only associated with Sec2p but they also carry the mRNA that encodes Sec2p and this association is regulated by Sec2p phosphorylation at residue S584.

## Results

### Sec2p is associated with mRNA

**Figure 1:**
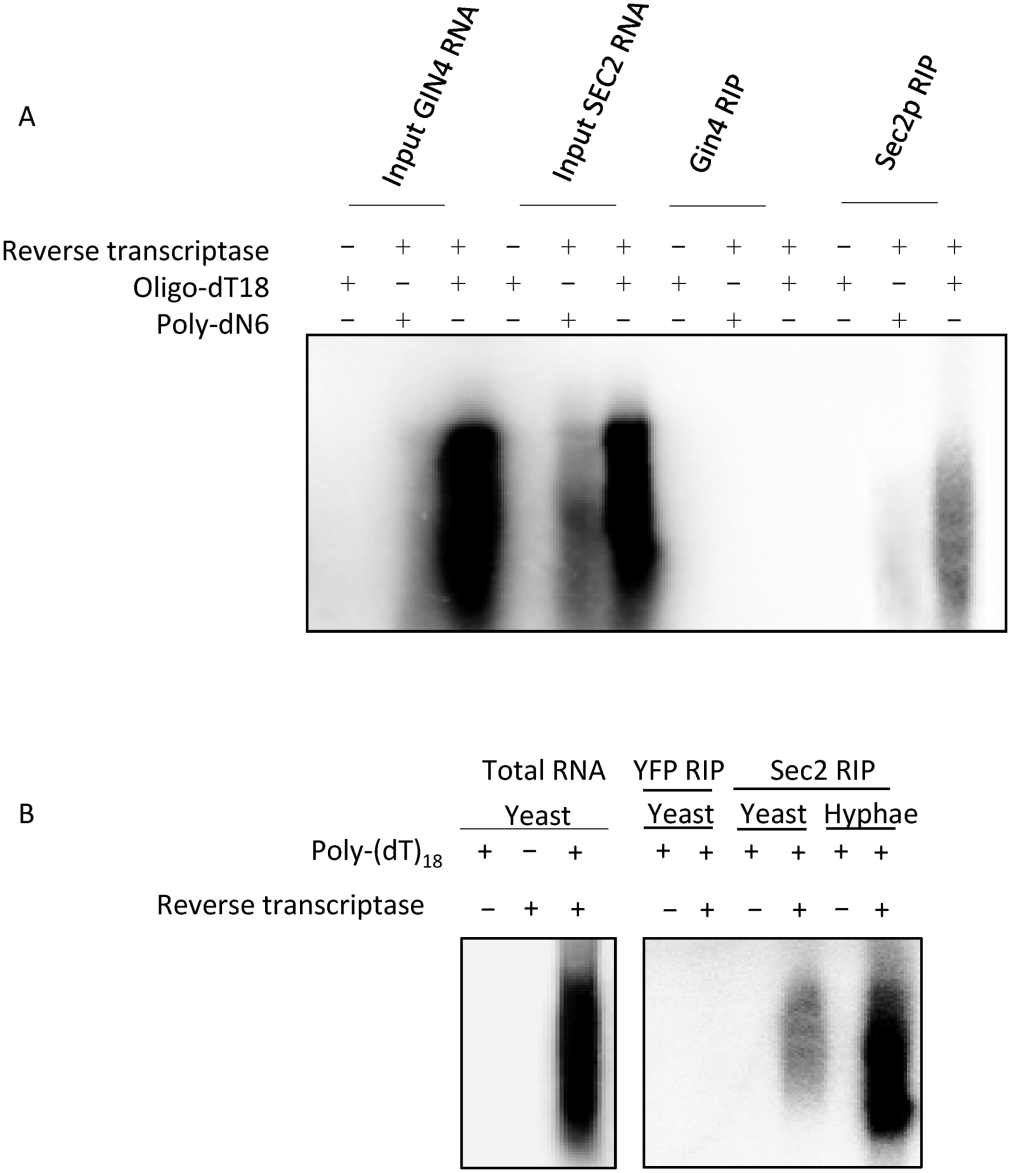
Sec2p is associated with polyA^+^ RNA and is more strongly associated in hyphae compared to yeast. A. Strains expressing Sec2p–YFP or control Gin4p–GFP as indicated were used in an RNA immune precipitation experiment (RIP). Any mRNA that was present in the immune precipitates was used as a template for RT PCR using either polydT (18) or randomized polyN6 primers, 32-P dCTP and Superscipt III reverse transcriptase as described in *Experimental procedures*. B. An RIP experiment using lysates from Yeast YFP expressed from the constitutive *PGK*1 promoter as a negative control and randomized Poly-(dT)_18_ as primers.

To investigate whether mRNAs that encode proteins localized to the tip are themselves asymmetrically localized we used Sec2p in an RNA Immune Precipitation (RIP) experiment as an example of a protein showing localization the Spitzenkörper of *C. albicans* hyphae. We immune precipitated Sec2p–YFP from hyphal and yeast cells using a monoclonal antibody against GFP and Gin4p–GFP, which is not polarized to the tip, as a control. The immune precipitates (IPs) were then incubated with ^32^P-labelled dCTP, Oligo dT and reverse transcriptase. Figure 1A shows that the RIP from Sec2p–YFP, but not Gin4p–GFP programmed the synthesis of cDNAs. In a separate experiment we investigated whether there was a difference in hyphae compared to yeast (Fig. 1B). In this experiment we used immune precipitated YFP from cells expressing YFP from the *PGK1* promoter as a control. The signal was clearly stronger from the hyphal sample compared to the yeast sample. These RIP experiments clearly show that mRNA molecules are associated with Sec2p and suggest that this association is greater in hyphal cells compared to yeast cells.

### The mRNA associated with Sec2p is *SEC2* mRNA

**Figure 2:**
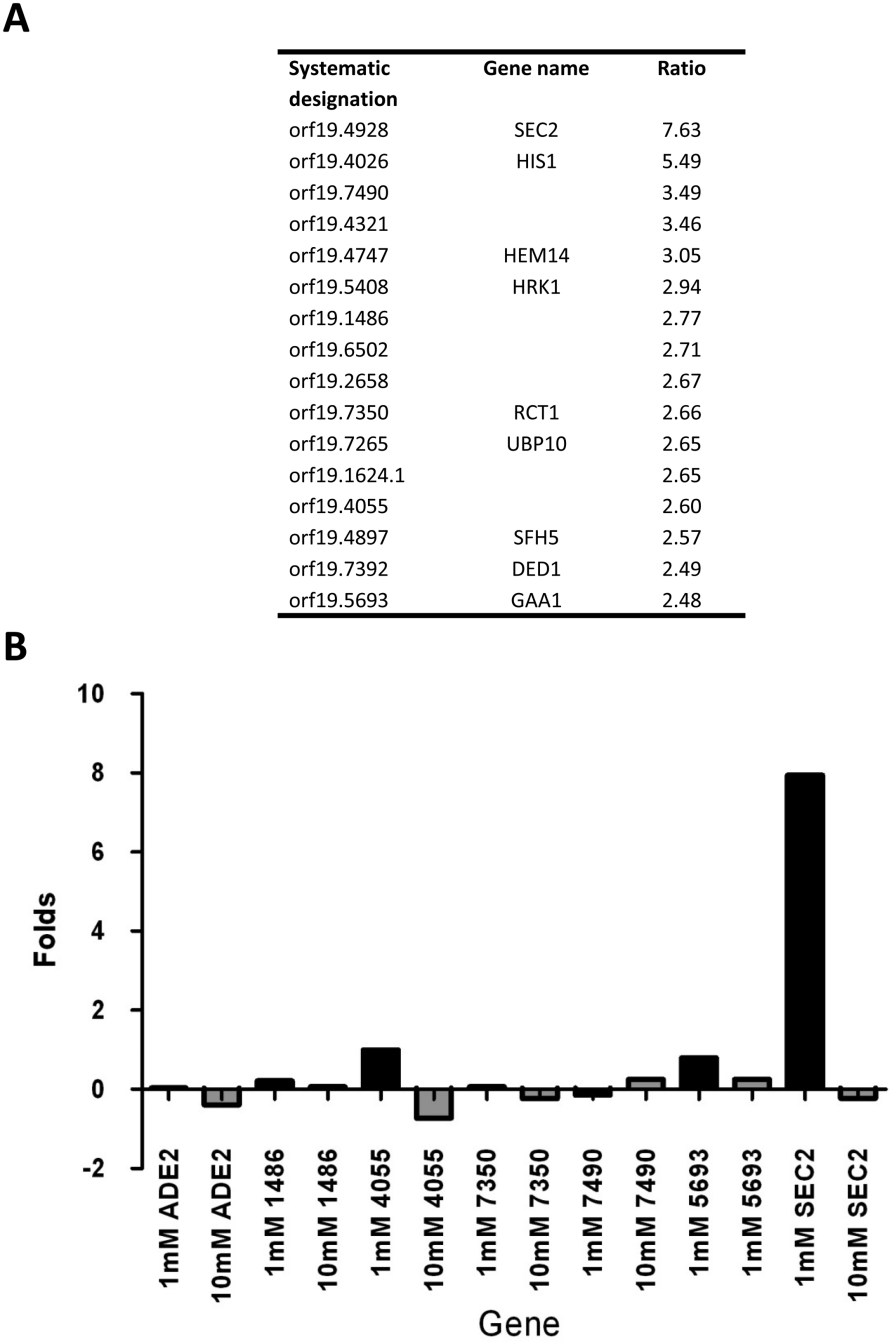
Sec2p–GFP is specifically associated with *SEC2* mRNA. A. The top 16 hits from a microarray analysis of the Sec2p-3xFLAG RIP experiment. The data are presented as the ratio of the indicated RNA to the control Gin4p-3xFLAG RIP. B. Verification of the microarray data for *SEC2* RNA and representative genes shown in panel A. qRT-PCR was performed using templates for the indicated genes when the lysis buffer contained either 1 mM EDTA or 10 mM EDTA to remove magnesium ions generally required for protein–RNA interactions. The gene names are shown in abbreviated form with the orf19 prefix not shown. The ordinate shows the fold increase relative to control Gin4p-3xFLAG RIP.

**Figure 3:**
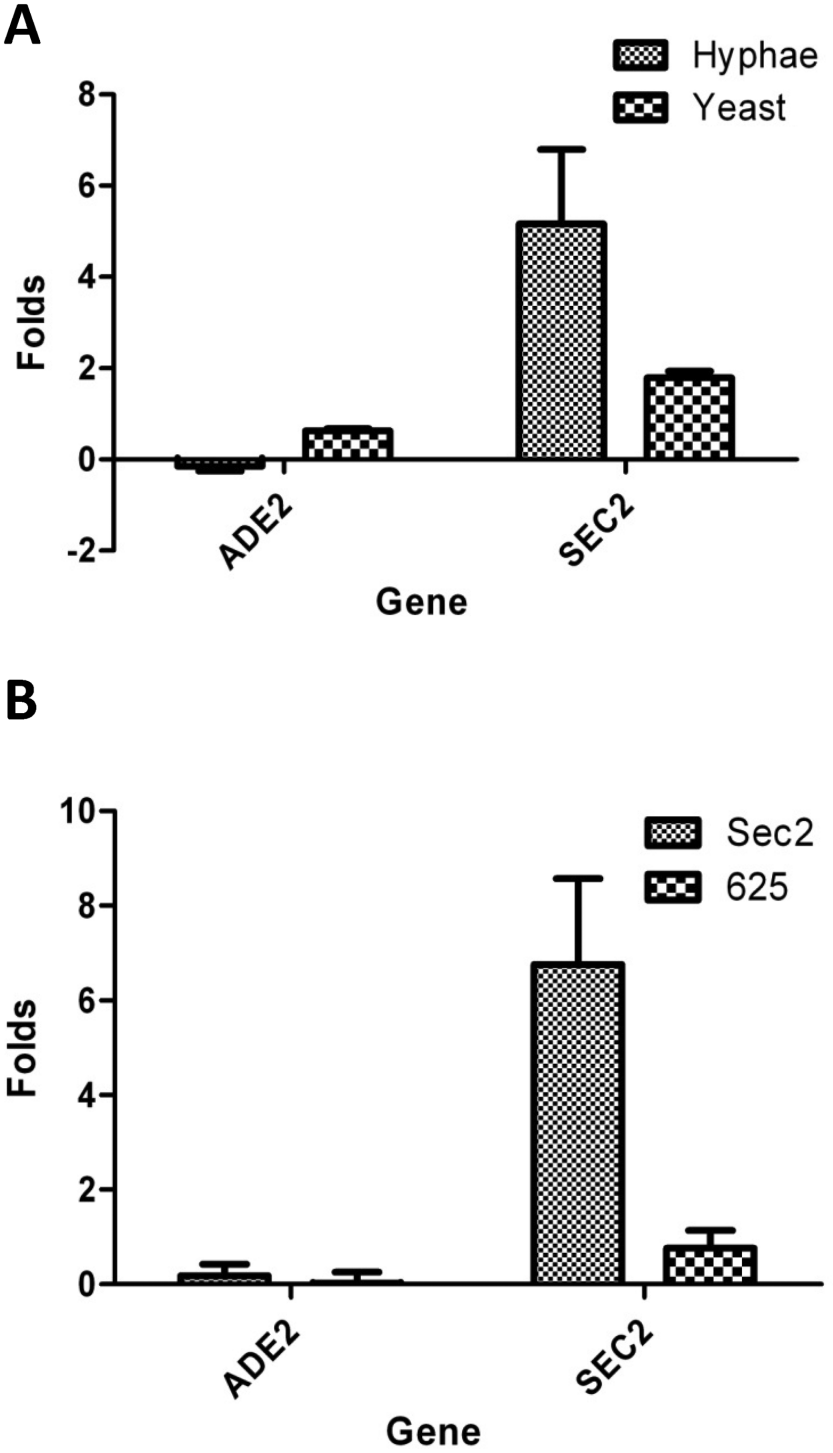
Sec2p shows a greater affinity for its mRNA in the hyphal form and requires the C-terminal domain for this affinity. A. A RIP experiment showing the fold increase of *SEC*2 RNA associated with Sec2p-3xFLAG in hyphae compared to yeast. B. A RIP experiment showing the fold increase of *SEC*2 RNA relative to Gin4p–GFP associated with full-length Sec2p–GFP (Sec2p_1–751_) compared to C-terminally truncated Sec2p (Sec2p_1–625_). Both experiments show the results of three biological replicates each with two technical replicates. In each case *ADE2* was used as a negative control.

In order to identify the mRNAs associated with Sec2p we hybridized the cDNA derived from a Sec2p-3xFLAG IP to a genomic microarray using Gin4p-3xFLAG as a control. The top 10 hits from this microarray are shown in Fig. 2A. Remarkably, the top hit was Sec2p itself with a ratio compared to the Gin4p-3xFLAG control of 7.63. The next hit was *HIS*1 with a ratio to Gin-3xFLAG of 5.49. Since the Sec2p-3xFLAG strain was *HIS*1 whereas the Gin4p-3xFLAG strain was Δ*his*1 this result shows that the enrichment of *SEC2* mRNA in the IP compared to the control is as least as great as the dynamic range of the microarray. In order to verify the presence of *SEC2* mRNA and to investigate whether the remaining hits were genuine, we carried out qRT-PCR on the Sec2p-3-FLAG IP using primers to *SEC*2 and to a selection of five of the remaining hits. *SEC*2 mRNA was consistently found to be enriched in the IP (Fig. 2B). In contrast, none of the other apparent hits from the microarray showed enrichment in qRT-PCR experiments. Figure 2B also shows that the association between the *SEC2* mRNA and Sec2p is dependent on Mg2^++^. Thus, Sec2p is associated only with its own encoding mRNA. Moreover, although there is a small enrichment in IPs from yeast cells, the enrichment in hyphae is up to three times greater, showing that the association of Sec2p mRNA with Sec2p is a feature of the strongly polarized growth of hyphae (Fig. 3A). The function of the C-terminal domain of Sec2p remains unknown. To determine if the C-terminal domain is required for binding *SEC2* mRNA we repeated the RIP experiment Sec2p_1–625_ which lacks 126 C-terminal residues. The resulting qRT-PCR shows a significant reduction in *SEC2* mRNA binding compared to control full-length Sec2p. This results show that either the C-terminal residues of Sec2p or the corresponding region of the encoding mRNA are required for binding (Fig. 3B).

### The association of *SEC2* mRNA with Sec2p is independent of She3p-based transport mechanism

**Figure 4:**
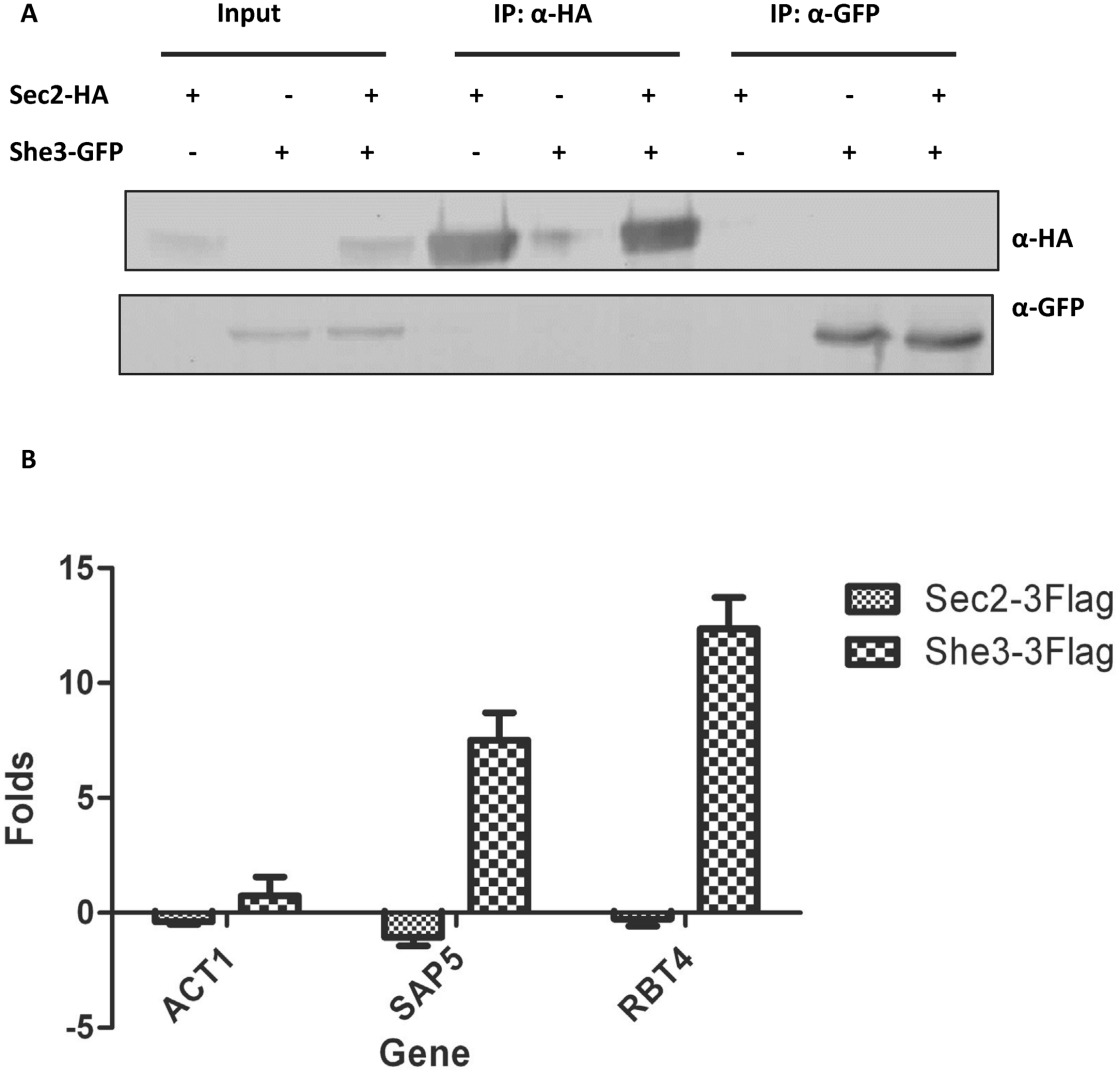
Sec2p is not associated with mRNAs transported by She3p.A. A reciprocal immune precipitation experiment showing a lack of detectable association between She3p and Sec2p. Sec2p-HA or She3p–GFP were immune precipitated as indicated (IP). The immune precipitates were used in a Western blot experiment with monoclonal antibodies to GFP or HA as indicated. B. RIP experiments were carried out with She3p-3xFLAG, Sec2p-3xFLAG and Gin4p-3xFLAG. RT-qPCR was carried out using primers to *ACT*1, *SAP*5 or *RBT*4. The chart shows the enrichment of the indicated RNAs in each immune precipitate relative to the Gin4p-3xFLAG control.

Previously a set of 40 mRNAs has been reported to be transported in a She3p-dependent manner in *C. albicans* hyphae (Elson *et al.*, 2009). However, *SEC*2 mRNA was not a member of this set. In that study, the criteria for selecting these 40 mRNAs was that they were in the top 5% of genes showing an enrichment when the mRNAs present in a She3p RIP were analysed by a microarray experiment. It is possible that *SEC*2 mRNA was enriched, but at a level below this threshold. We therefore inspected the online data file of the microarray and found that *SEC*2 mRNA showed no enrichment. To investigate this further, we determined whether She3p and Sec2p physically associate in a reciprocal co-immunoprecipitation experiment (Fig. 4A). No association was observed. Next we determined whether Sec2p associates with *SAP*5 and *RBT*4 mRNAs that have been shown to be associated with She3p (Elson *et al.*, 2009). We confirmed the enrichment of both of these mRNAs in a She3p IP, but there was no enrichment of these mRNAs in a Sec2p IP (Fig. 4B). We conclude that the association of *SEC2* mRNA with Sec2p is independent of the She3p-based mRNA transport mechanism reported previously (Elson *et al.*, 2009).

### Sec2p colocalizes with *SEC2* mRNA

**Figure 5:**
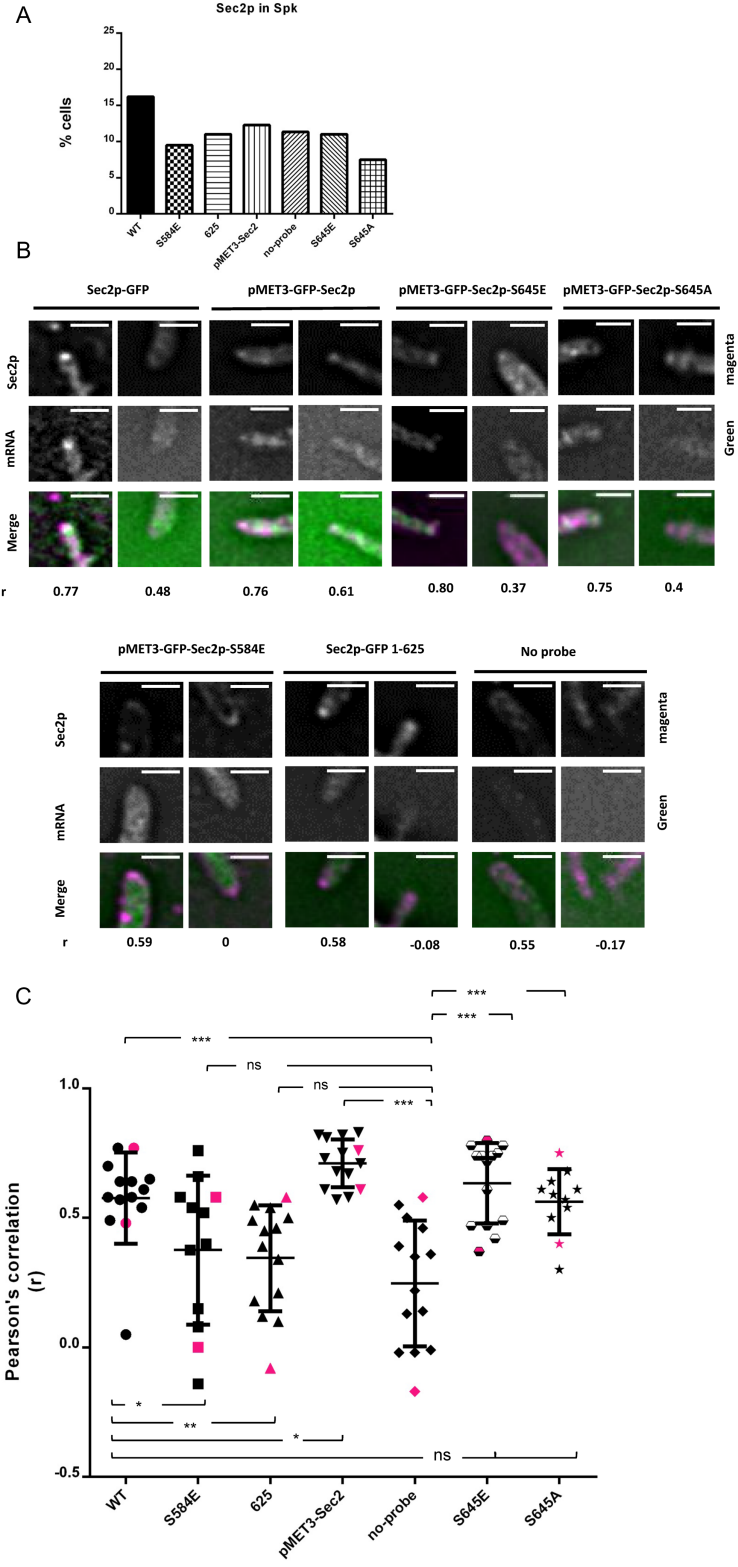
*SEC2* mRNA colocalizes with Sec2p. Unbudded stationary-phase cells of the indicated genotype were induced to form hyphae for 90 min and briefly fixed with 2% v/v formaldehyde as described in the text and *Experimental procedures*. In the resulting hyphae Sec2p–GFP or GFP–Sec2p was visualized by immunocytofluorescence with a monoclonal anti-GFP antibody and a secondary antibody conjugated to red Alexafluor 633 (red); mRNA was visualized in the same hyphae by *in situ* hybridization to a panel of oligonucleotides conjugated to FITC (green). A. Percentage hyphal tips showing apical localization of the GFP signal. B. Two hyphal tips from each strain showing mRNA and GFP localization chosen to show the range of colocalization observed. The merged image is shown with the red signal from the GFP immunofluorescence false coloured magenta. With this green-magenta combination, which aids the colour-blind, colocalized signals appear white. The Pearson correlation coefficient (*r*) of colocalization is shown below each hypha. Scale bars: 5 microns. C. Pearson correlation coefficients from all hyphae analysed in each strain. The magenta symbols indicate the correlation of the hyphae shown in panel B. Tests of significance between the data sets are shown by the horizontal brackets; *** indicates *P* ≤ 0.0005; ** indicates *P* ≤ 0.005; * indicates *P* ≤ 0.05; ns indicates not significant.

In order to establish whether the association of Sec2p with its mRNA occurs *in vivo* we sought to colocalize Sec2p with its mRNA. We initially employed the MS2 system in which *SEC2* mRNA was fused to a sequence containing 24 MS2 binding sites in a cell expressing MS2-binding protein fused to GFP. However, we were unable to detect a specific *SEC2* mRNA signal. We next turned to a fluorescence *in situ* hybridization (FISH) strategy in which we colocalized the signal from Sec2p–GFP in the Spitzenkörper with the signal from the *in situ* hybridization of a panel of 10 oligonucleotides probes conjugated to FITC. This approach was complicated by the effect of fixation on Sec2p localization. At least in *C. albicans,* the Spitzenkörper is not an organelle-like structure with a fixed organization. Rather it appears to be an accumulation of secretory vesicles. Upon the fixation necessary for FISH these vesicles along with Sec2p are dispersed. To address this problem we progressively reduced both the fixation time and the concentration of the formaldehyde. We found that fixation for 5 min in 2% v/v formaldehyde resulted in the preservation of apical Sec2p localization in approximately 10–15% of the hyphae (Fig. 5A). A second problem is that the fluorescence of GFP is lost during FISH, so to localize Sec2p–GFP we used immunocytofluorescence with the secondary antibody fused to Alexofluor 633. This red fluorophore also allowed us to distinguish between the signals from FITC and GFP which would otherwise both have been in the green channel. We examined hyphae showing apical Sec2p–GFP localization to see if the mRNA signal from FISH colocalized with the Sec2p–GFP signal in the Spitzenkörper. We quantified any colocalization of the two signals using the Coloc2 plugin from the Fiji implementation of NIH ImageJ (*Experimental procedures*) that generates a Pearson correlation score (*r*), where a score of +1 indicates a perfect correlation. As a negative control we omitted the hybridization probe. Figure 5B shows the mRNA and Sec2p signals from two hyphal tips selected to show the range colocalization association observed. In the merged images the Sec2p signal is false coloured magenta so that areas of colocalization appear white. Figure 5C shows the *r* scores from all of the individual hyphal tips examined along with the mean and the standard deviation of the *r* scores. To show how the visual signals of colocalization are reflected in the *r* scores, the *r* scores from the actual hyphal tips shown in Fig. 5B are displayed with a magenta symbol in Fig. 5C. The data confirm that Sec2p–GFP colocalizes with its mRNA and that the mean of *r* scores is significantly different from the negative control and the non-binding Sec2p mutants described below.

As well as examining the colocalization of Sec2p–GFP and its mRNA we also examined the colocalization of *SEC2* mRNA in the following *SEC2* alleles: First, in our original study of the role of phosphorylation in *SEC2* function we constructed a number of alleles using the regulatable *MET3* promoter to allow their conditional expression. We wished to test whether some of these alleles affected Sec2p binding to its mRNA. To do this it was first necessary to establish that the C-terminal GFP–Sec2p fusion bound its mRNA in the same way as the Sec2p–GFP N-terminal fusion. Figure 5B and C shows that GFP–Sec2p expressed from the *MET3* promoter did indeed clearly colocalize with its mRNA. Note that this was a heterozygous construct so that there was a second wild-type *SEC2* allele present. The slightly stronger and more consistent colocalization of *GFP–SEC2* compared to *SEC2–GFP* may be due to slight overexpression of GFP–*SEC2* from the *MET3* promoter, and/or to the presence of two *SEC2* alleles expressing mRNA in contrast to the single *SEC2–GFP* allele. Second, S645 is located in a region of low complexity which potentially may be part of an mRNA-binding domain. Recently it has been shown that RNA-binding proteins are enriched in low complexity regions and that phosphorylation of these regions reduces affinity of these proteins for RNA (Han *et al.*, 2012). To test whether phosphorylation of S645 affects the affinity of Sec2p for its mRNA we used hemizygous phosphomimetic and non-phosphorylatable substitutions at this position (GFP–Sec2-S645E/*sec2*Δ and GFP–Sec2-S645A/*sec2*Δ). We found both forms of Sec2p colocalized with GFP–*SEC2* mRNA in a nearly identical fashion to wild-type Sec2p–GFP (Fig. 5B and C). Importantly, because this strain was hemizygous, the only *SEC*2 mRNA species in these cells lacks the native 5′ UTR, thus the affinity of Sec2p for its mRNA does not require the 5′ UTR. Third, the phosphomimetic GFP–Sec2p-S584E failed to colocalize with its mRNA confirming the reduced affinity of this protein for its mRNA in the biochemical tests described above (Fig. 5B and C). Lastly, *SEC2*_1–625_–GFP also failed to colocalize with its mRNA, again confirming the reduced affinity of this C-terminally truncated protein for its mRNA in the biochemical tests (Fig. 5B and C).

### Sec2p mRNA is associated with secretory vesicles

**Figure 6:**
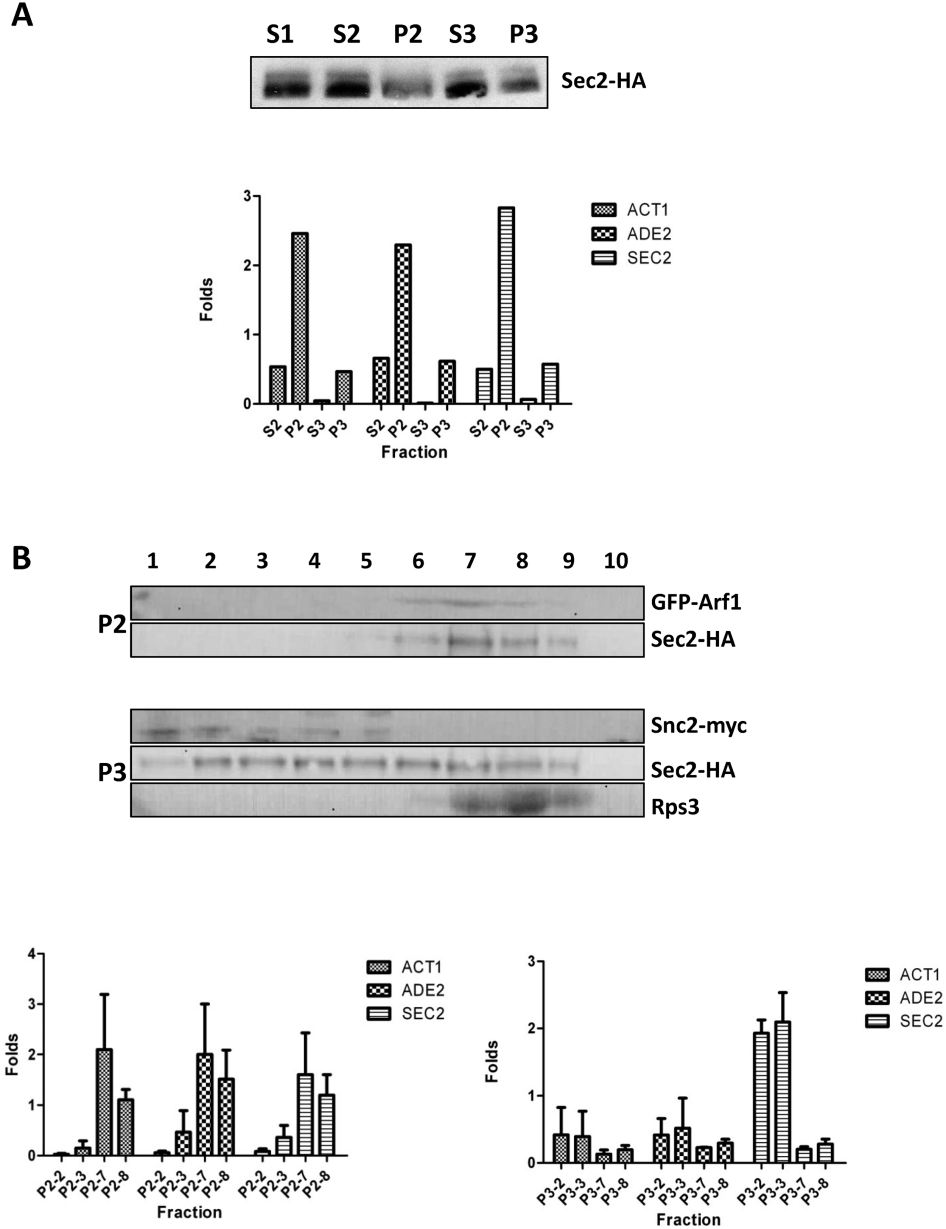
*SEC2* mRNA co-fractionates with secretory vesicles. A. Hyphal cells were harvested 90 min after induction and fractionated as described in *Experimental procedures*. Sec2p-HA was quantified by Western blot (top). *ACT*1, *ADE1* and *SEC2* mRNAs were quantified by qRT-PCR and fold enrichment compared to the concentration of each in the S1 supernatant. S1 = 10 000 g supernatant, S2 = 30 000 g supernatant, S3 = 100 000 g supernatant. P2 = 30 000 g pellet, P3 = 100 000 g pellet. B. The P2 and P3 fractions were resuspended and further fractionated in an Optiprep density gradient. Fractions were collected and Arf1p–GFP, Sec2p-HA and the ribosomal protein Rps3p were detected by Western blotting. *ACT*1, *ADE1* and *SEC2* mRNAs were quantified by qRT-PCR and fold enrichment compared to the concentration of each in the S1 supernatant. Data shown are the mean and SEMs of three separate biological experiments.

One possible explanation for the association of *SEC2* mRNA and its encoding protein is that IP is pulling down nascent Sec2p as it emerges from ribosomes. A precedent for this is the association of Abp140p with polyribosomes as it is translated on actin cables (Trautwein *et al.*, 2004). Moreover, if the phenomenon was not due to the IP of nascent Sec2p, it was of interest to discover whether the association was occurring with the cytosolic pool of Sec2p or with Sec2p associated with secretory vesicles. To address both of these questions we followed the distribution of Sec2p-HA and *SEC*2 mRNA in a cell fractionation experiment. Cells were fractionated to produce 10 000 g, 30 000 g and 100 000 g pellets (P1–P3) and corresponding supernatants (S1–S3) (Fig. 6A). The P2 and P3 pellets were resuspended and further fractionated by Opitprep density centrifugation as previously described (Chang *et al.*, 2008) (Fig. 6B). GFP–Arf1p was used to provide a Golgi marker [we verified that GFP–Arf1p has an intracellular distribution similar that shown for other Golgi proteins in *C. albicans* (Rida *et al.*, 2006)]; Snc1p-Myc was used to provide a secretory vesicle marker; and an antibody to Rps3p was used to identify the fractions containing ribosomes (Frey *et al.*, 2001). qRT-PCR was used to quantify *SEC*2 mRNA; *ADE*2 and *ACT*1 mRNAs were quantified as controls.

The results show that Sec2p-HA is distributed in the S1–S3 supernatants and the P2 and P3 pellets (Fig. 6A). Sec2p-HA in the S3 fraction represents the cytosolic pool. *SEC*2 mRNA was found in both the S2, P2 and P3 fractions, but not in the S3 fraction (Fig. 6A). Thus *SEC2* mRNA is not associated with the cytosolic pool of Sec2p. The density centrifugation of the resuspended P2 and P3 pellets show that Sec2p-HA co-fractionates with the Golgi marker GFP–Arf1p, the secretory vesicle marker Snc2p-myc, and to a more limited extent with the ribosomal marker Rps3p (Fig. 6B). Since secretory vesicles are derived from the Golgi, the association of Sec2p-HA with both Golgi-containing and secretory vesicle-containing fractions is to be expected. The overlapping distribution of Sec2p with ribosomal fractions may represent nascent Sec2p. The *SEC2* mRNA and the control *ADE*2 and *ACT1* mRNAs are enriched in fractions P2-7 and P2-8 which co-fractionate with the Golgi marker, Arf1p–GFP (Fig. 6B). Importantly, in three independent experiments *SEC2* mRNA was consistently enriched in the P3-2 and P3-3 fractions which co-fractionate with the secretory vesicle marker Snc2p-myc, whereas there was no such enrichment of either of the control *ACT*1 or *ADE2* mRNAs (Fig. 6B). Thus *SEC*2 mRNA, is associated with the secretory vesicle fraction but the control mRNAs are not. Moreover, there is no enrichment of *SEC*2 mRNA in the P3-7 and P3-8 fractions that overlap with the Rps3p positive fractions (Fig. 6B). Thus, the *SEC*2 mRNA that associates with the Sec2p is not due to the association of nascent protein with its mRNA on polyribosomes as it is translated.

### Sec2p physically associates with mRNA

**Figure 7:**
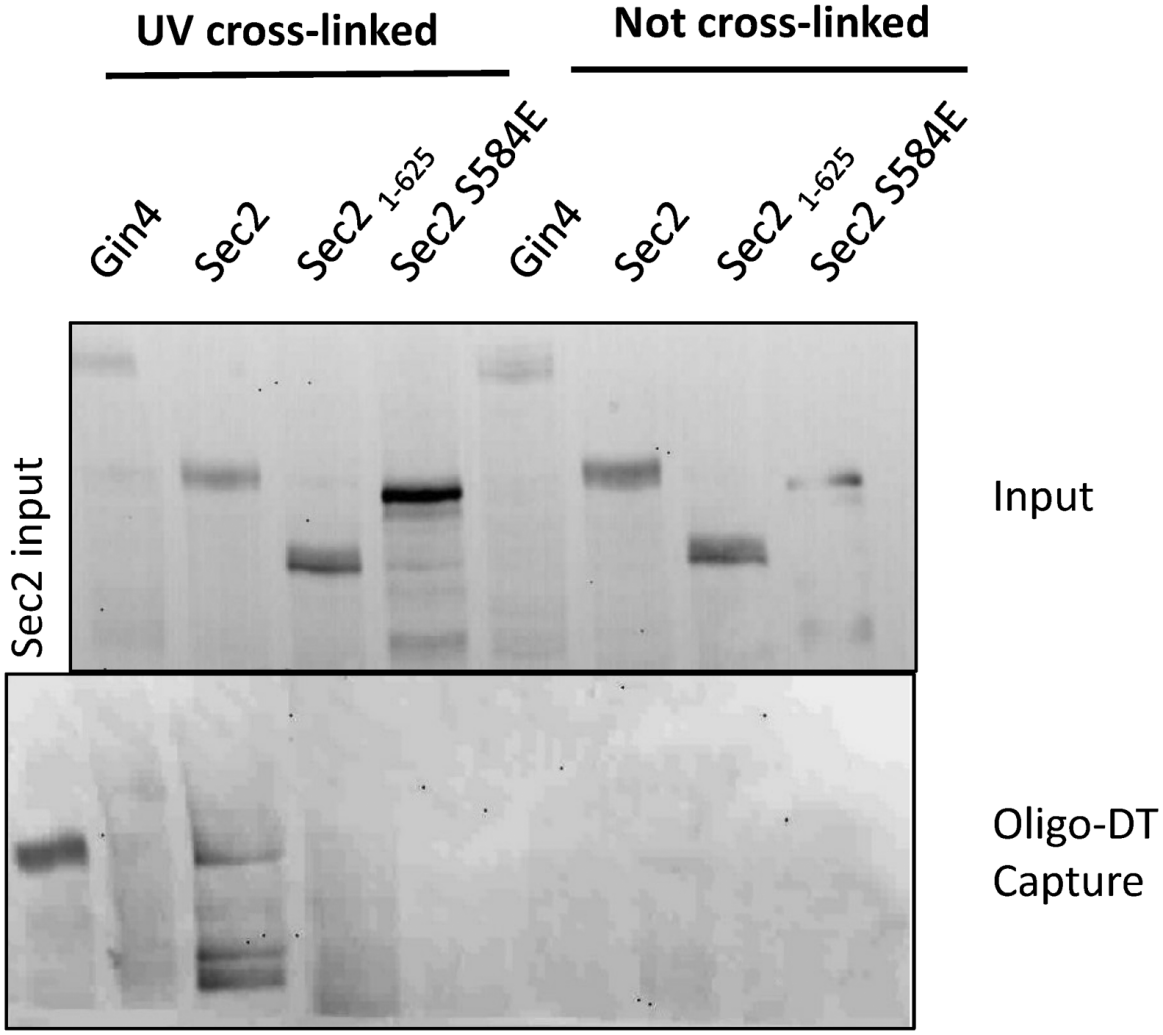
*SEC2* mRNA is physically associated with Sec2p. Gin4p–GFP, Sec2p–YFP, Sec2_1–625_ and GFP–*SEC2* S584E as indicated were used in an RNA capture experiment as described in *Experimental procedures*. Membranes from the input and oligo-DT captured samples were probed with a monoclonal antibody to GFP.

The association of *SEC2* mRNA with secretory vesicles could be due to a direct physical association with Sec2p. Alternatively both *SEC2* mRNA and Sec2p could be independently carried by secretory vesicles without any direct physical interaction. In this latter scenario the Sec2p RIP also pulls down secretory vesicles resulting in the apparent association of Sec2p and *SEC2* mRNA. To address this question we carried out an RNA capture experiment. Lysates from a cell expressing either Sec2p–YFP, Sec2_1–625_ or GFP–Sec2p S584E were UV irradiated to cross-link proteins to RNA and DNA. Cellulose-Oligo dT was then used to capture mRNA before proteins associated with the mRNA were analysed by Western blotting using a monoclonal antibody to GFP. Gin4pGFP was used as a negative control. The results show that Sec2p, but not Gin4p, directly associates with mRNA (Fig. 7). Moreover, the Sec2_1–625_ truncation show no association, consistent with the reduction of the Sec2_1–625_ association with *SEC*2 mRNA observed in RIP experiment (Fig. 3B). We previously showed that phosphorylation of a serine at residue 584 is necessary for Sec2p to support hyphal growth. GFP–Sec2p S584E showed no association with mRNA, even though it was expressed at a higher level than Sec2p which showed an association. This result suggests that the role of S584 phosphorylation is to programme the release of *SEC2* mRNA.

## Discussion

Previous work in *S. cerevisiae* has shown that mRNAs for polarity proteins are localized to the tips of small buds and colocalize with the encoded protein (Aronov *et al.*, 2007). Moreover, this mRNA localization is depended on proteins involved in the late secretory pathway such as the GTPases Cdc42p and Rho3p. the exocyst components Sec10p and Sec15p, the Rab GTPase Sec4p and the v-SNARE components Sncp1/2 and the t-SNARE Sec9p (Aronov and Gerst, 2004). These observations led us to investigate the possibility that the mRNAs are transported on secretory vesicles. However, since polarized growth is restricted to a short period of the *S. cerevisiae* cell cycle, we used hyphae of the human fungal pathogen *C. albicans* which shows continuous polarized growth throughout the cell cycle. We immune precipitated Sec2p the GEF for Sec4p. We found evidence that mRNAs were associated with Sec2p, but remarkably, only *SEC*2 mRNA was present the immune precipitate and the association was independent of She3p. Sec2p is associated with vesicles, but upon vesicle tethering to the exocyst, it is thought to be displaced into the cytosol where the bulk of Sec2p is found (Medkova *et al.*, 2006; Novick *et al.*, 2007). Cell fractionation experiments showed that compared to control mRNAs, *SEC*2 mRNA is over-represented in the vesicle and Golgi fractions,. Thus, our experiments show that Sec2p is associated with its own mRNA as it travels on secretory vesicles. To our knowledge, the identification of GEF as an RNA-binding protein is unique among fungi. However, several Arf GTPases have been shown to bind mRNA in *S. cerevisiae* (Trautwein *et al.*, 2004; Scherrer *et al.*, 2010) and in mammalian cells, Arf-GEF1 was identified as a candidate RNA-binding protein in the Atlas of mRNA-binding proteins (Castello *et al.*, 2012).

### Examples of mRNA protein association in fungi

As well as the transport of *ASH*1 mRNA and mRNAs encoding polarity proteins by She2p/She3p/Myo4p-based process that has been extensively studied in *S. cerevisiae*, there are several other cases of mRNA transport that have been studied in fungi. In hyphae of the corn smut fungus *Ustilago maydis*, the RNA-binding protein Rrm4p has been shown to associate with a set of 63 mRNAs that are transported bidirectionally on microtubules by the motor proteins the dynein Dyn1p, and the kinesin Kin1p (König *et al*., 2009; Baumann *et al.*, 2012). Rrm4p was shown to recognized CA-rich sequences in the 3′ UTR. Rrm4p RNPs colocalize with the endosomal protein Yup1 suggesting that Rrm4p RNPs are associated with endosomes which are also transported bidirectionally on microtubules by Dyn1p and Kin1p. Recently it has been shown that the early endosomes are associated with ribosomes. Significantly, the association of ribosomes with early endosomes is impaired in an *rrm4* mutant and inhibitors of protein translation (Higuchi *et al.*, 2014). Together these observations suggest that Rrm4p acts to load mRNAs onto early endosomes where they are translated. Such a mechanism may ensure that translation is equally distributed throughout the hypha. It has also been shown that septin RNA and septin protein are transported on the same endosomes, and that this is necessary for the correct delivery of septins to the cell poles (Baumann *et al.*, 2014). This provides an interesting parallel to the present study – in both cases a protein and its encoding mRNA are transported on vesicles to their site of localization. In *C. albicans* 40 mRNAs have been shown to be associated with She3p (Elson *et al.*, 2009). However, apart from *ASH1*, there was limited overlap with the gene set that associates with She3p in *S. cerevisiae*. This She3p-based mRNA transport is physiologically important as *she3*ΔΔ cells were defective invasive growth and epithelial cell damage. Although *in situ* hybridization showed that some of the She3p-associated transcripts were enriched in apical regions, none showed the specific localization to the Spitzenkörper that we have observed in the case of *SEC2.*

Our results show significant differences with this previous work as well as the She3p-based system in *S. cerevisiae* and *C. albicans.* First, the affinity of Sec2p for its own mRNA does not involve She2p/3p or any other previously characterized RNA-binding protein or motif in the RNA. We discuss below the way in which Sec2p may recognize its mRNA. Second, the affinity of Sec2p for its own mRNA is unusual in that it appears that only a single mRNA is bound, whereas most RNA-binding proteins recognize multiple RNAs. Third, the presence of polyribosomes and thus mRNA in the fungal Spitzenkörper has been known for some time, but our work for the first time establishes the identify of an mRNA localized in the Spitzenkörper.

### How does Sec2p recognize bind to its mRNA?

In *S. cerevisiae* the preferred motifs for a number of RNA-binding proteins have been elucidated (Hogan *et al.*, 2008). Many of these are located in either the 5′ or 3′ UTR. The *SEC2-3xFLAG* proteins we used in the RIP experiments, and the Sec2p–GFP we used in the colocalization experiments, lacked the native 3′ UTR and there was no other *SEC2* mRNA species present which carried a 3′ UTR. Both of these proteins bound *SEC2* mRNA, thus the interaction does not require the 3′ UTR. The colocalization experiments showed that both GFP–Sec2p S645E and GFP S645A proteins colocalized with *SEC2* mRNA. Again there was no *SEC2* mRNA species present which carried a 5′ UTR, thus the 5′ UTR is also not required for the interaction. The possibility that the *SEC2* mRNA does not directly physically interact with Sec2p, but rather both are carried on secretory vesicles is eliminated by the RNA-capture experiments which demonstrate that Sec2p cross-links to oligo-dT purified mRNA. However, the S584E allele which mimics phosphorylation abolishes the interaction of Sec2p for its mRNA, while a C-terminal truncation Sec2_1–625_ reduces the interaction. These observations suggest Sec2p region between 584 and 625 may be involved in the binding its mRNA.

### The cellular location of the Sec2p interaction with its mRNA

It is difficult to envisage that Sec2p can shuttle between the hyphal tip and nucleus. However, recognition of cytosolic proteins for their cognate mRNAs has precedents. For example the survey of RNA binders in *S. cerevisiae* described above, found that 30% of RNA-binding proteins tested bound their own mRNAs, and many of these proteins were cytosolic (Scherrer *et al.*, 2010). A similar finding was reported in survey of the RNAs bound by known RNA-binding proteins in *S. cerevisiae* (Hogan *et al.*, 2008). Thus mechanisms must exist for the association of proteins and their cognate mRNAs. Many mRNAs encoding cytosolic proteins have been found to localize to the ER (Lerner *et al.*, 2003). Moreover, the GTPase Arf1p, which is required for vesicle formation in the Golgi, has been found to associate with the mRNA-binding protein, Pab1p. This association was shown to be mRNA dependent. *ASH*1 was found to one of the associated mRNAs and its association was independent of She3p (Trautwein *et al.*, 2004). However, asymmetric localization of *ASH1* mRNA was abolished in an *arf1* mutant. Interestingly, one of the mRNAs found to associate with Arf1p was the actin-binding protein *ABP140*, which was shown to localize to the distal pole of the mother cell (Kilchert and Spang, 2011). Localization of Abp140p mRNA is an interesting example of a protein that binds its own mRNA, because the first 17 amino acids of Abp140p forms an actin-binding motif; while the first 67 amino acids are sufficient to bind mRNA. Thus, it is proposed that as Abp140p is translated, the nascent polypeptide binds actin as it emerges from the polyribosome and thus tethering the polyribosome to actin cables for transport to the distal pole. Forcing the ectopic localization of Abp140p to different cellular locations resulted in the corresponding mislocalization of *ABP140* mRNA. The above examples illustrate that mRNA association with proteins occurs with the ER, Golgi and on actin cables. Sec2p is a protein that cycles between the Golgi and the hyphal tip along actin cables. Figure 6 shows that *SEC2* mRNA is detected in both Golgi and secretory vesicle fractions. We suggest that *SEC2* mRNA is loaded onto vesicles in the Golgi before onward transport to the hyphal tip.

### Possible roles of *SEC2* association with its mRNA

An interesting role for the association of proteins with their cognate mRNAs has been demonstrated in the cases of mammalian thymidylate kinase (TS) and dihydofolate reductase (DHFR) which associate with their own cognate mRNAs and repress their own translation (Tai *et al.*, 2004). In *S. cerevisiae* Khd1p binds to *ASH1* mRNA and represses its translation (Paquin *et al.*, 2007). Interestingly, phosphorylation of Khd1p by the casein kinase Yck1p, removes Khd1p from *ASH1* mRNA and releases the translational repression. These examples suggest one possible role of Sec2p association with its mRNA is autoregulation of its translation which is relieved by phosphorylation of S584. This argument is supported by observation that the Spitzenkörper is rich in polyribosomes suggesting that it a centre for protein translation (Grove *et al.*, 1970).

Asymmetric mRNA localization has been associated with localization of the encoded protein. Such mRNA localization mechanisms often act in parallel with other mechanisms of protein localization. For example, in *S. cerevisiae* proteins encoded by the mRNAs transported by the She3p system still localize in the absence of She3p, including proteins localized to sites of polarized growth (Shepard *et al.*, 2003; Aronov *et al.*, 2007). If the association of Sec2p with its encoding protein is promotes localization of Sec2p to the Spitzenkörper, then this may also be act in parallel with other localization mechanisms because the GFP–Sec2p_GFP 584E and Sec2p_1–625_–GFP still localize to the Spitzenkörper even though the mutations abolish the association with mRNA. However, the situation may be more complex than this. A C-terminal truncation, Sec2p_1–583_ that deletes the phosphorylation site causes mis-localization of Sec2p and abrogates hyphal growth (Bishop *et al.*, 2010).

In *S. cerevisiae* it has been shown that both Ypt32p and Sec15p bind Sec2p in overlapping regions and thus must compete with each other for Sec2p binding (Ortiz *et al.*, 2002; Medkova *et al.*, 2006; Novick *et al.*, 2007). Ypt32p is a Rab GTPase that acts to load Sec2p on nascent secretory vesicles in the trans-Golgi; while Sec15p is an exocyst component to which secretory vesicles are tethered prior to fusion with the plasma membrane. It has been proposed that displacement of Ypt31p by Sec15p upon vesicle docking allows Sec2p to exit the secretory vesicle upon docking to be available for another round of vesicle transport from the Golgi (Novick *et al.*, 2007). An interesting possibility in *C. albicans* is that phosphorylation of Sec2p on S584 releases its binding to mRNA on the vesicles in the Spitzenkörper to allow it to be recycled to the Golgi.

## Experimental procedures

### Media and growth conditions

Cultures were routinely cultured on YEPD consisting of 2% glucose, 2% Bacto peptone and 1% Bacto yeast extract (Difco Becton, Dickinson and Company New Jersey) plus 80 mg l^−1^ uridine. SD medium consists of 0.67% w/v yeast nitrogen base (Difco, Becton, Dickinson and Company, New Jersey), 2% w/v glucose, 80 mg l^−1^ each of uridine or 40 mg l^−1^ histidine, and arginine. To induce hyphal growth cells were grown to saturation at 30°C in YEPD medium. An aliquot of the saturated culture was inoculated into fresh YEPD plus 10% calf serum (Sigma-Aldrich, St Louis, USA) so that the resultant OD_600_ = 0.5. The cells were then incubated at 37°C.

### Strains and plasmids constructions

Strains constructed are listed in Table S1, and the oligonucleotides used are listed in Table S2. All strains were derived from BWP17 (Wilson *et al.*, 1999). Gene deletions and C-terminal YFP and HA fusions were performed as previously described (Gola *et al.*, 2003; Walther and Wendland, 2003; Schaub *et al.*, 2006; Lavoie *et al.*, 2008). All strains were checked for correct genome integration by PCR. Correct expression of protein fusion strains were also checked by Western blot. 3xFLAG fusion proteins were constructed as above using the plasmid pFA 3xFLAG *Ura3* which constructed as follows: 3xFLAG was amplified by PCR from plasmid p3xFLAG-Myc-CMV26 (Sigma Aldrich) using primers 3xFLAG-F(PstI) and 3xFLAG-R. The fragment was subcloned into pGEM T easy (Promega, Madison, USA*)* then extracted using PstI and BamHI and cloned into pFA *URA*3. This plasmid was then used to create 3xFLAG fusion proteins as described above.

### Protein extracts and Western blotting

Total protein extracts were prepared with modified RIPA buffer containing phosphatase inhibitors (50 mM Tris-HCl pH 7.2, 0.1% Sodium deoxycholate, 0.1% Triton X-100, 50 mM NaF, 0.2 mM sodium orthophosphate, 0.2 mM β-glycerol-phosphate, 100 mM NaCl, and protease inhibitors tablet (Roche Biosciences, Lewes, UK) and 200 μl of glass beads (0.4 mm; Sigma-Aldrich, St Louis, USA) were added. Cells were broken for 30 s in a minibeadbeater (Biospec Products, Bartlesville, USA). Soluble proteins were obtained by centrifugation of total extracts at 13 400 r.p.m. over 10 min at 4°C. For Western blots, 30 μg of protein extracts were separated on 6% SDS-PAGE, transferred to Hybond-P membranes (GE Healthcare, Chalfont St Giles, UK), and probed with anti-GFP monoclonal antibody (Roche Biosciences, Lewes, UK) anti-HA (Secondary antibodies conjugated to horseradish peroxidase were diluted 1:5000.

### RNA-IP and microarray analysis

Cells from an overnight culture were diluted in YEPD and incubated for 4 h at 30°C or YEPD plus serum and incubated for 90 min at 37°C. Cells were spun down and wash once with water and once with lysis buffer (50 mM HEPES pH7.5, 100 mM NaCl, 1mMEDTA, 1 mM DTT, 10% glycerol, 0.5% Triton X-100, 6 mM MgCl_2_, 1 mM PMSF, plus Protease Inhibitors tablet). Cells were disrupted in lysis buffer containing 200 units RNase inhibitors (Ribosafe Bioline, London) and 0.2 μg ml^−1^ of heparin using a minibeadbeater (Biospec Products, as above). The lysate was cleared by centrifugation. To quantify input RNA 50 μl was removed from the lysate and analysed using RNAeasy mini kit (Qiagen, Taunton, MA ). Three millilitres containing 3 mg ml^−1^ of total protein was inmunoprecipitated using protein G Sepharose pre-incubated with α-FLAG monoclonal (Sigma-Aldrich) or α-GFP monoclonal antibody (Roche Biosciences, Lewes, UK). The resin was washed three times with lysis buffer without protein inhibitors. The RNA was then eluted with 0.12 μg μl^−1^ of Proteinase K at 37°C for 45 min. The RNA was purified using RNAqueos micro kit (Ambion, Austin, USA). Microarray analysis was carried out by NRC Biotechnology Research Institute, Montreal, Canada.

### RT-qRT-PCR

Reverse transcription was carried out using Superscript III (Invitrogen, Carlsbad, CA, USA) following the manufacturer's instructions. The cDNA was diluted 1:5 and qRT-PCR was carried out following the manufacturer's instructions from Sensi –mix Syber green Kit (Bioline) in a Corbett Research Rotor gene qPCR machine. The primers used for the amplification are listed in Table S2. Normalization was done using the ΔΔCt method for two technical replicates and three biological replicates.

### Ribonucleoparticle (mRNP) capture

An overnight culture was diluted into 400 ml of fresh medium plus 10% Calf serum (Sigma Aldrich, St Louis, USA) and incubated at 37°C. After 90 min of hyphal induction cells were harvested, washed with PBS and transferred to a 12 cm Petri dish floating in an ice-water bath and irradiated with UV light at a distance of 10 cm from the light source [Spectrolinker XL-1500 (254 nm), DOT Scientific, Burton, MI, USA] three times for 2.5 min each time. Irradiated cells were pelleted and resuspended in lysis buffer (50 mM HEPES pH 7.5, 100 mM NaCl, 1 mM EDTA, 1 mM DTT, 10% glycerol, 0.5% Triton X-100, 6 mM MgCl_2_, 1 mM PMSF, plus Protease Inhibitors tablet) containing 200 units RNase inhibitors (Ribosafe, Bioline), and disrupted using a minibeadbeater (Biospec Products). The lysate was cleared by centrifugation. Three milligrams of total protein was denatured by adding an equal volume of 2× binding buffer (20 mM Tris-HCl pH 7.5, 1 M NaCl, 1% SDS, 0.2 mM EDTA) and then incubated with 0.01 g of oligo-dT cellulose for 2 h at room temperature. Oligo-dT cellulose was washed three times with 50% v/v mixture lysis/binding buffer. The proteins were eluted with 60 μl of elution buffer (10 mM Tris-HCl pH 7.5, 1 mM EDTA, 50 μg ml^−1^ RNase) and incubated at 37°C for 30 min.

### Cell fractionation

Cell fractionation and iodixanol density gradients were carried out as previously described (Chang *et al.*, 2008) with the lysis buffer described above. Western blots were performed as described (Bishop *et al.*, 2010). An antibody against the Rps3p ribosomal protein of *S. cerevisiae* was used to identify ribosomal fractions (Frey *et al.*, 2001). Because it was not possible to combine all the epitope fusions into a single strain the experiment shown in Fig. 6 was repeated out using either a GFP–*ARF1 SEC2*-HA strain or a *SNC*2-MYC *SEC2*-HA strain. The data shown are a composite of the GFP–Arf1 and Sec2p-HA signals from an experiment using the *GFP–ARF1 SEC2-HA* strain and the Snc2-myc from an experiment using the *SEC*2-HA *SNC*2-*MYC* strain (where the Sec2p-HA signal was identical to that shown). An identical result was obtained when the combination of strains was reversed.

### Combined immunocytochemistry and FISH

The immunocytochemistry was modified from Sudbery (2001). An overnight yeast culture was diluted into 25 ml of SD medium with 10% of calf serum to an OD_600_ of 0.5. Hyphal induction was carried out at 37°C for 90 min. The culture was fixed in 2% formaldehyde for 5 min. To separate cell clumps, cells were pelleted by centrifugation and washed 3× in 55 mM HCl and finally resuspended in 500 μl of 5 mg·ml^−1^ of pepsin in 55 mM HCl and incubated for 15 min at 37°C with gentle shaking. The cells were pelleted by centrifugation and washed 3× in buffer B (1.2 M Sorbitol, 100 mM potassium phosphate buffer pH 7.5) and finally resuspended in 1 ml spheroplasting buffer [1× Buffer B, 20 mM Vanadyl ribonucleoside complexes solution (Sigma-Aldrich), 5 μl β-Mercaptoethanol, and 1600 units of Lyticase (Sigma-Aldrich)] to digest the cell wall for 30 min at 37°C. The digested cells were washed once in buffer B and resuspended in 500 μl of buffer B. A 20 μl sample of these spheroplasts was pipetted into one well of a multi-well slide (Hendley, Essex, UK) and allowed to stand for 30 min at 4°C. All the liquid was removed by aspiration and the slides were washed in methanol at −20°C for 5 min and acetone at −20°C for 30 s. The slides were air dried. Each well was washed 10× in blocking buffer (1× PBS, 2 mg ml^−1^ BSA). Then, 20 μl of primary antibody (mouse anti-GFP, Roche), diluted 1:100 in blocking buffer, was added to the well. The slides were incubated overnight in a humid chamber at 4°C. The well was then washed 5× with blocking buffer plus 0.1% v/v Triton X-100, and 5× in blocking buffer. Then, 20 μl of secondary goat anti-mouse antibody conjugated with Alexafluor-633 (Life Technologies) diluted 1:250 was add to each well and incubated for 1 h at room temperature. The wells were then washed 5× in blocking buffer plus 0.1% Triton X-100, and 5× in blocking buffer. The antibodies were fixed for 5 min in blocking buffer plus 2% v/v of formaldehyde. Followed by two washes with 2× SCC for 5 min and finally 1× with 20% formamide in 2× SSC, 0.1% v/v Triton X-100 for 10 min. The FISH probe was prepared as follows: 10 ng·μl^−1^ of every probe (10 probes in total) labelled with Fluorescein (Sigma-Aldrich) were mixed with 1 μg·μl^−1^ ssDNA, 1 μg·μl^−1^ tRNA in solution 1 (40% Formamide, 10 mM Sodium phosphate buffer (pH 7), then denatured at 95°C for 5 min and mixed with an equal volume of solution 2 (4× SCC, 20 mM vanadyl ribonucleoside, 4 μg/μl BSA). Twenty microlitres of this mixture was added to every well and the slides were incubated in a humid chamber at 37°C for 3 h in the dark. Then, cells were washed 2× with: 20% formamide, 2× SSC for 10 min at 37°C, 1× with: 2× SSC, 0.15% Triton X-100 for 10 min at room temperature 2× with 1× SSC, for 10 min at room temperature and 2× with 1× PBS for 5 min at room temperature. Finally the slides were allowed to dry in the dark and the coverslips were mounted with mounting solution, consisting of 80% glycerol in 1× PBS containing 5 μg·ml^−1^ of DAPI.

### Colocalization analysis

Only cells that showed at Sec2p localized to a Spitzenkorper-like structure were analysed. Colocalization of the red (protein) and green channel (mRNA) in hyphal tips was analysed using the Coloc2 plugin for the Fiji implementation of ImageJ (http://fiji.sc/Fiji) to generate a Pearson's correlation coefficient (*r*).

### Microscopy and live cell imaging

Widefield epifluorescence microscopy and differential interference contrast (DIC) microscopy was carried out using a Delta Vision RT microscope (Applied Precision Instruments, Seattle) using an Olympus 100× UplanAPo NA 1.35 lens (Olympus Tokyo, Japan). Images were acquired and deconvolved with Softworx™ software. Images are maximum intensity projections of the deconvolved Z-stack unless otherwise stated. To visualize the nucleus DNA was stained with DAPI (Sigma-Aldrich, St Louis) To visualize the cell outline in fluorescent images, cells were counterstained with 1 μg·ml^−1^ Calcofluor White (Fluorescent Brightener 28, Sigma Aldrich). Where indicated cells were fixed with 2% formaldehyde and then treated with pepsin as previously described (Sudbery, 2001).

### Statistical analysis

Graph pad Prism 5 was used to analyse and to represent all the data shown in this work.
