# Supplementary material for: In Candida albicans hyphae, Sec2p is physically associated with SEC2 mRNA on secretory vesicles
Source: Mol Microbiol. 2014 Oct 14;94(4):828–42. doi: 10.1111/mmi.12799 (PMC4278529; doi:10.1111/mmi.12799)
Supplement: Supplementary file 1 — Supporting information [file mmi0094-0828-SD1.pdf]

Table S1 Strains used in this study

Sheffield strain number	Relevant genotype	Full genotype	Source
305	*BWP17*	*URA3*::*λimm434/URA3*::*λimm434his1*::*hisG/his1*:: *hisG arg4*::*hisG/arg4*::*hisG*	(Wilson et al. 1999)
805	*SEC2 YFP*	*sec2::HIS1/SEC2 YFP::URA3*	(Bishop et al. 2010)
835	*URA3-pMET3-YFP::SEC2/SEC2*	*BWP17 URA3::pMET3YFP::SEC2/SEC2*	(Bishop et al. 2010)
838	*SEC2-625 YFP*	*sec2::HIS1/SEC2(1-625) YFP::URA3*	(Bishop et al. 2010)
1049	*GIN4*-GFP	BWP17 *GIN4-GFP*::*URA3*	(Wightman et al. 2004)
1051	*GIN4*-3FLAG	*GIN4*/ *GIN4*-3xFLAG::URA3	This study
1154	*pMet3-YFP-sec2-S584E/ sec2Δ*	*BWP17 URA3-pMet3-YFP-sec2-S584E-ARG4/ sec2::HIS1*	(Bishop et al. 2010)
1156	*pMet3-YFP-SEC2-S645E/ sec2Δ*	*BWP17 URA3::Met3-YFP-Sec2-S645E::ARG4/ sec2::HIS1*	(Bishop et al. 2010
1164	*pMet3-YFP-SEC2-S645A/ sec2Δ*	*BWP17 URA3::pMet3-YFP-Sec2-S645A::ARG4/ sec2::HIS1*	(Bishop et al. 2010)
1329	*ARF1 GFP*	*ARF1/ARF1 GFP ARG4*	This Study
1331	*ARF1 GFP/ SEC2 HA*	*sec2::HIS1/SEC2 HA::URA, ARF1/ARF1 GFP ARG4*	This Study
1365	*SEC2 3xFLAG*	*sec2::HIS1/SEC2 3FLAG::URA3*	This Study

Table S2 Primers used in this study

**PRIMER**	**Nucleotide sequence (5' → 3')**
S1 SEC 3xFLAG	ATTTAAAGATCAATTAAATAAAGAATTAGATCAAACTTTAGAGATGTTAGCCGAAAATATTGATTTTGATGAGAGTAGTAATGGTAATGGTAATGGTAT**ATGGACTACAAAGACCATGACGGT**
S1 SEC2 HA	ATTTAAAGATCAATTAAATAAAGAATTAGATCAAACTTTAGAGATGTTAGCCGAAAATATTGATTTTGATGAGAGTAGTAATGGTAATGGTAATGGTAAT**GGTCGACGGATCCCCGGGTA**
S2 SEC2	TAACGACAGCAATGGGCCCAGAAAATCCCGTCAAAAAAAAATCCACAATACGCGTAATGCACTCTAGAATTTCGTAAATGATTCATTCATAATTAACAAATCTGATATCATCGATGAATTCGAG**TCGATGAATTCGAGCTCGTT**
S1 ARF1 XFP	TCCAAGCTACTTGTGCCACTACTGGGGATGGTTTATACGAAGGTTTGGAATGGTTATCTACTAACTTGAAAAACTCTTCA**GGTGCTGGCGCAGGTGCTTC**
S2 ARF1 XFP	TCCACCAGTAAAATAATAATAAAGATAAGAGGTGAAGTGGTGAAGTGGTGAAGTGGGAACAATATTAGAGAGGGAAAACA**TCTGATATCATCGATGAATTCGAG**
S1 GIN4 3xFLAG	TAAAGTTACAACTGATACTTTATTTTCTGAAATTGAAAAGGTCTTACTCAAAGAAGGTGTTTTAGATAAAATGGACTACAAAGACCATGACGGTG
S2 GIN4 3xFLAG	TGCTTTTATTAGCATGTTTACTCAATGGGGGGTTGGAAGCTTTACTGATACTGTTGGACCTACTTATATATCGATGAATTCGAGCTCGTT
G1-SEC2	CTGAACCCGTACAAGAACCAG

G1-ARF1	GGGATGTCGGTGGTCAAGAT
3xFLAG-R	GGCCGCAAGCTTGTCATCGT
URA3-F	GGAAGAGATCCAGATATTGAAGG
URA3-R	TGTGCTACTGGTGAGGCATG
ARG4-F	CTGCTAAAAGTGCCGTTTTAAAACAATT
ARG4-R	ACCGGTGAAACGACCACCCC
HIS1-F	GGACGAATTGAAGAAAGCTGGTGCAACCG
HIS1-R	GGGATATCAGCTGCAGGCAAGAAGATTAAT
YFP-R	GGATAAGGCAGATTGGTAGG
pCaHA-3	TTAACCGGCATAGTCTGGGA
Sec2-R157-Flc	GTTCTGCATCTGAATCCTCT
Sec2-R541-Flc	GGCTACGTATATCTCCTTGT
Sec2-R800-Flc	AGCAGACGATGAAGATACCG
Sec2-R1043-FLc	CGAGGCAATTACATGAGTGT
Sec2-R1091-Flc	GTCAGATTGTTGCCACTGT
Sec2-1284-Flc	CCAGAGGCATTGTTACCCTT
Sec2-R1417-Flc	TGGTAGCAGGTAAAGAAGGG
Sec2-R1715-Flc	ATTGGTATGAATGCTATTGG
Sec2-R1958-Flc	GGCTTCATCAAATAACGAGG
Sec2-R1941-Flc	TTGCTAACCATGGTGTTGGC
ADE2-F1	AAGGAATCTCCATTGGTGGG
ADE2-R1	GGCCGCCACCATACCTGGCA
ACT1-F	GGCCGCCACCATACCTGGCA
ACT1-R	AGCAATACCTGGGAACATGG
SEC2-F1	CCTAAACCAACCCAACAACAA
SEC2-R1	TTCTGCTGATGCTGTTTCTGA
1486-F2	GCCATCATTGGTGTCACCTT
1486-R1	TGATTTGGTGGTATTGGTGGT
4055-F1	CTGCCCTAGCCAGGGTTAAT
4055-R1	GCAAATACTGCAACGACCAA
5693-F1	AGCCCTTCCATTGACATTTG
5693-R1	ACCACCCCAAAACAACGATA
7350-F1	TATCTACTGTCGCCGGTGGT
7350-R1	CCACCATATTGTTGCTCGTG
7490-F2	ATCAAACGTCAGGCATTGGT
7490-R1	TGCATAACCAATATCCATACTCCA

References

Bishop, A., Lane, R., Beniston, R., Lazo, B., Smythe, C., and Sudbery, P.2010. Hyphal growth in *Candida albicans* requires the phosphorylation of Sec2 by the Cdc28-Ccn1/Hgc1 kinase. *EMBO J*.**29:**2930-2942.

Wightman, R, Bates, S, Amnorrattapan, P, and Sudbery, P. E.2004. In *Candida albicans,* the Nim1 kinases Gin4 and Hsl1 negatively regulate pseudohypha formation and Gin4 also controls septin organization. *J Cell Biol*.**164:**581-591.

Wilson, B, Davis, D, and Mitchell, A. P.1999. Rapid hypothesis testing in *Candida albicans* through gene disruption with short homology regions. *J Bacteriol*.**181:**1868-1874.
